# Reliability and validity of ultrasonography in evaluating the thickness, excursion, stiffness, and strain rate of respiratory muscles in non-hospitalized individuals: a systematic review

**DOI:** 10.1186/s12903-023-03558-y

**Published:** 2023-12-02

**Authors:** Emma FengMing Zhou, Siu Ngor Fu, Chen Huang, Xiu Ping Huang, Arnold Yu Lok Wong

**Affiliations:** Department of Rehabilitation Sciences, The Hong Kong Polytechnic University, Hong Kong, China

**Keywords:** Diaphragm, Low back pain, Inspiratory muscle, Stiffness, Ultrasound, Shear wave elastography

## Abstract

**Objective:**

To summarize the reliability and validity of ultrasonography in evaluating the stiffness, excursion, stiffness, or strain rate of diaphragm, intercostals and abdominal muscles in healthy or non-hospitalized individuals.

**Literature search:**

PubMed, Embase, SPORTDiscus, CINAHL and Cochrane Library were searched from inception to May 30, 2022.

**Study selection criteria:**

Case–control, cross-sectional, and longitudinal studies were included if they investigated the reliability or validity of various ultrasonography technologies (e.g., brightness-mode, motion-mode, shear wave elastography) in measuring the thickness, excursion, stiffness, or strain rate of any respiratory muscles.

**Data synthesis:**

Relevant data were summarized based on healthy and different patient populations. The methodological quality by different checklist depending on study design. The quality of evidence of each psychometric property was graded by the Grading of Recommendations, Assessment, Development and Evaluations, respectively.

**Results:**

This review included 24 studies with 787 healthy or non-hospitalized individuals (e.g., lower back pain (LBP), adolescent idiopathic scoliosis (AIS), and chronic obstructive pulmonary disease (COPD)). Both inspiratory (diaphragm and intercostal muscles) and expiratory muscles (abdominal muscles) were investigated. Moderate-quality evidence supported sufficient (intra-class correlation coefficient > 0.7) within-day intra-rater reliability of B-mode ultrasonography in measuring right diaphragmatic thickness among people with LBP, sufficient between-day intra-rater reliability of M-mode ultrasonography in measuring right diaphragmatic excursion in non-hospitalized individuals. The quality of evidence for all other measurement properties in various populations was low or very low. High-quality evidence supported sufficient positive correlations between diaphragm excursion and forced expiratory volume in the first second or forced vital capacity (*r* >  = 0.3) in healthy individuals.

**Conclusions:**

Despite the reported sufficient reliability and validity of using ultrasonography to assess the thickness, excursion, stiffness, and strain rate of respiratory muscles in non-hospitalized individuals, further large-scale studies are warranted to improve the quality of evidence regarding using ultrasonography for these measurements in clinical practice. Researchers should establish their own reliability before using various types of ultrasonography to evaluate respiratory muscle functions.

**Trial registration:**

PROSPERO NO. CRD42022322945.

**Supplementary Information:**

The online version contains supplementary material available at 10.1186/s12903-023-03558-y.

## Introduction

The diaphragm is a dome-shaped muscle that separates the thoracic and abdominal cavities [1]. In addition to being the principal inspiratory muscle that contributes to 70–90% of tidal volume in different positions [2], the diaphragm also plays an essential role in the visceral system as well as the musculoskeletal system. Specifically, it involves in the functioning of various internal organs such as emesis, urination, defecation, and the prevention of gastroesophageal reflux [3–5]. Further, the diaphragm harmoniously controls inspiration and postural control, stabilizes the lumbar spine, and contributes to optimal performance of daily activities or sports [6–9].

Because the diaphragm works synergically with parasternal and external intercostals to expand the rib cage during inspiration [10, 11], uncoordinated contraction of synergists can increase the work of breathing and increases the burden of the diaphragm [10]. Likewise, while abdominal muscles serve as the force-expiratory muscles when respiratory loading increases [10, 11], the tonic activity of abdominal muscles helps maintain the optimal length of diaphragm for better force generation during the inspiration in an upright position [12]. Therefore, it is essential to comprehensively evaluate various respiratory muscles (e.g., intercostals and abdominal muscles) by reliable objective assessments in order to better assess diaphragmatic function in individuals, and to inform clinical decision-making.

Ultrasonography (USG) is a non-invasive in vivo ultrasound imaging approach to evaluate the morphometry, function, or mechanical properties of soft tissues with different imaging modes [13, 14]. Prior research has used brightness-mode (B-mode) and motion-mode (M-mode) USG to assess the thickness and excursion of diaphragm, respectively in critically ill patients (e.g., ventilated patients) in order to estimate the inspiratory function of diaphragm [15, 16]. Diaphragm thickness fraction as measured by B-mode USG is used as a predictor for successful weaning in ventilated patients [13, 14, 17]. Although previous systematic reviews have supported the reliability and validity of B-mode USG in assessing the morphometry of diaphragm in ventilated patients [18, 19], their findings cannot be generalized to non-hospitalized individuals given the diverse functions of diaphragm in different conditions. Additionally, although some studies have used B-mode and M-mode USG to investigate the morphometry and mobility of intercostals and abdominal muscles in different populations [20–22], no systematic review has summarized the reliability or validity of such USG in these respiration-related muscles in non-hospitalized individuals.

Ultrasound shear wave elastography (SWE) is another type of USG that has recently been used to measure respiratory muscle stiffness [23–26]. SWE is an objective, and reproducible method to quantify the mechanical properties of soft tissues [27, 28], although there are some concerns regarding the validity of using SWE to measure biomechanical properties of the diaphragm [29]. Given the controversy, it is important to conduct a systematic review to summarize the reliability and validity of SWE in measuring respiratory muscle stiffness.

Against this background, the current systematic review aimed to summarize the evidence regarding the reliability and validity of various types of USG (including SWE) in evaluating the thickness, excursion, stiffness, or strain rate of diaphragm, intercostal muscles or abdominal muscles in non-hospitalized patients and healthy individuals.

## Methods

This review protocol was registered with PROSPERO (CRD42022322945) and was reported according to the guidelines of Preferred Reporting Items for Systematic Reviews and Meta-Analyses [30].

### Literature search

PubMed, Embase, SPORTDiscus, CINAHL and Cochrane Library were systematically searched from inception to May 30, 2022 to identify relevant studies without language restrictions. The main keywords were reliability, validity, ultrasonography, shear wave elastography, and respiratory muscles. Relevant search strings with Boolean operators and linking terms were used (Supplementary File-S 1). Forward citation tracking of the included studies was conducted using Scopus. Backward citation tracking was also conducted. The corresponding authors were contacted by emails for additional relevant articles.

### Eligibility criteria

Case–control, cross-sectional, and longitudinal studies were included if they investigated the reliability or validity of various types of USG in measuring the morphometry, function, or mechanical properties of any respiratory muscles. Animal and cadaveric studies, reviews, case reports, commentaries, and letters to the editors were excluded. Two reviewers (FZ and XH) independently performed title and abstract screening of the identified citations according to the selection criteria. Between-reviewer disagreements were reconciled by consensus, or by the jurisdiction of a third reviewer (AW). Relevant full-text articles were retrieved. The same procedure was repeated for the full-text screening. Between-reviewer agreements were evaluated by Kappa coefficients (κ).

### Data extraction

Two independent reviewers (FZ and CH) extracted relevant information from the included studies: (1) authors’ information (e.g., names, publication year, country); (2) study characteristics (e.g., study design, setting); (3) assessor’s information; (4) participants’ demographics (e.g., gender, age, types of population); (5) measurements (e.g., types of USG used and assessment locations); (6) outcomes (e.g., intra- or inter-rater reliability, which might be expressed as intra-class correlation coefficients (ICCs) or kappa coefficients and the respective 95% confidence interval (95%CI); and convergent/divergent validity). Any disagreements in data extraction were resolved by discussion or by the judgment of the third reviewer (AW).

### Quality assessments and quality of evidence

The Consensus-based Standards for the Selection of Health Measurement Instruments (COSMIN) checklist was used to evaluate the methodological quality of the included studies. Clinician‐Reported Outcome Measures (ClinROMs) checklist [31] was used to assess the quality of the included reliability studies. Patient‐Reported Outcome Measures (PROMs) checklist [32] was used to evaluate the quality of validity studies (using Boxes 9a and 9b to evaluate studies investigating convergent validity and discriminative/known-groups validity, respectively). The methodological quality of the included studies was rated as “very good, adequate, doubtful, or inadequate” using the “worst-score counts” principle [31, 32].

Against the updated criteria for good measurement properties [32] (Supplementary File-S 2), the reliability and validity of various types of USG in each included study was rated as sufficient (“ + ”), insufficient (“-”), or indeterminate (“?)”. Likewise, the overall quality of evidence for reliability and validity of various types of USG for a given muscle assessment was first checked against the criteria for good measurement properties [32] (Supplementary File-S 2) to determine the overall consistency of each measurement property as “sufficient ( +), insufficient (-), inconsistent ( ±), or indeterminate (?)”. Then, the quality of evidence for each measurement property in overall population and each subgroup (different populations in the included studies) was graded as “high, moderate, low, or very low” using the modified GRADE approach as suggested by COSMIN [32] (Supplementary File-S 3). These processes were conducted by two independent reviewers (FZ and CH). Any disagreements were resolved by discussion or by the judgment of a third reviewer (AW).

### Data synthesis

Data were categorized and analyzed according to different patient populations. Although meta-analysis using random effects models in RevMan 5 was planned, it was infeasible to conduct the meta-analysis because no outcome of interest was evaluated under the same condition (e.g., USG modes, probe locations, participants’ positions, breathing phases) in two or more studies. Therefore, a narrative review was conducted.

## Results

### Study selection

**Fig. 1:**
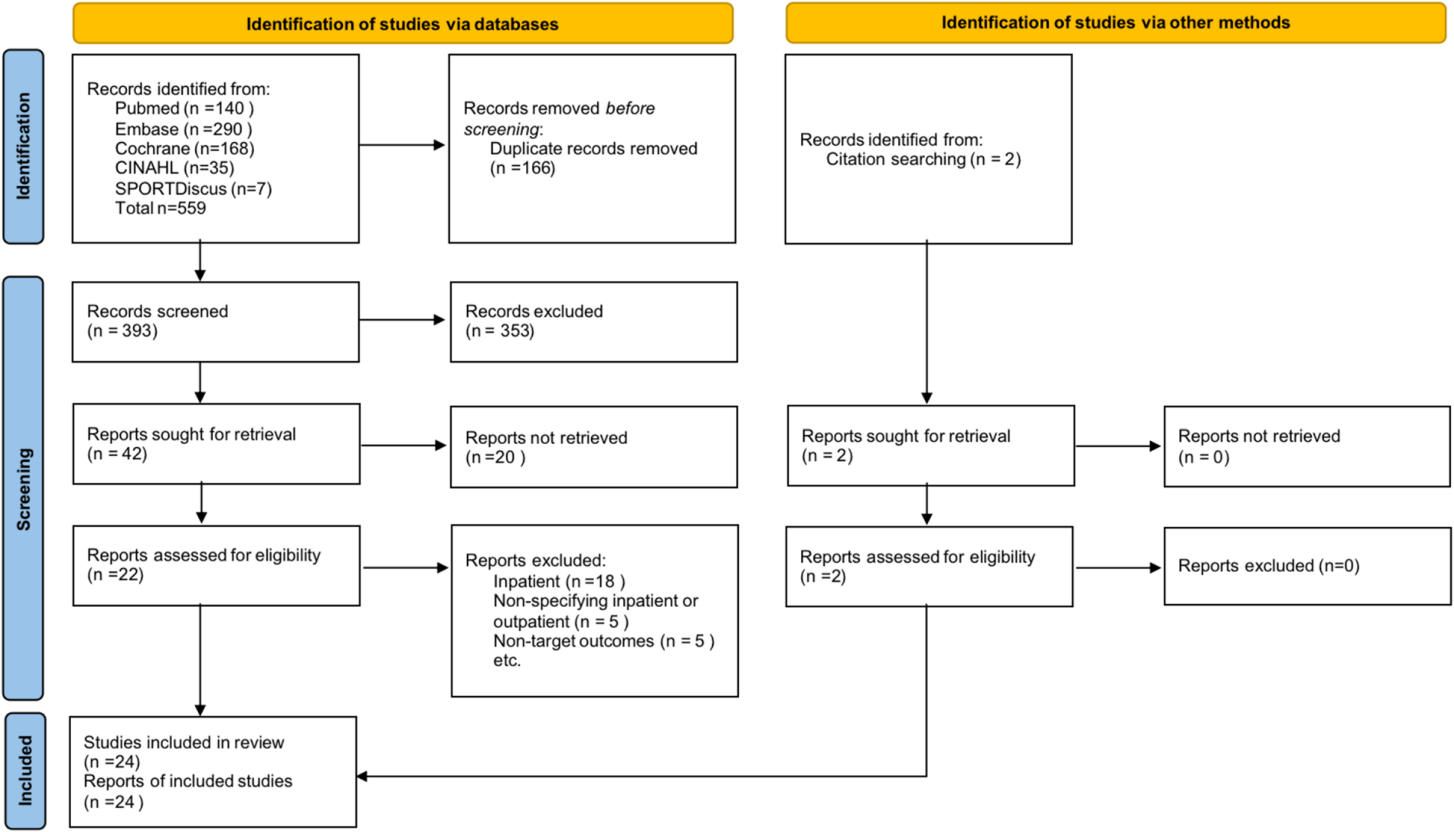
Flow chart of study selection inclusion into the systematic review

Of 1,110 identified citations from databases and other sources, 395 were included for the title and abstract screening after removing duplicates. Following the full-text screening, 24 articles were included (Figure 1). The inter-rater agreement for title and abstract and full text screening were good (κ = 0.88, 95%CI: 0.80 to 0.96) and adequate (κ = 0.73, 95%CI: 0.57 to 0.88), respectively [33].

### Study characteristics

The 24 included studies were published between 1998 and 2021 involving 787 participants (aged: 12–70 years) (Table 1). Twenty-one included studies reported reliability (20 on intra-rater and 13 on inter-rater reliability) and eight reported validity (6 on convergent validity and 3 on discriminative/known-groups validity). Four included studies involved people with LBP (*n* = 73, aged: 20–50 years), two involved people with chronic obstructive pulmonary disease (COPD) (*n* = 63, aged: 57–79 years), two involved teenagers with adolescent idiopathic scoliosis (AIS) (*n* = 48, aged: 12–17 years, Cobb angles ranging from 12**°**-47**°**), and the remaining studies involved healthy individuals (*n* = 603, aged: 11–70 years). Three respiratory muscles were investigated. Specifically, 19 included studies examined the diaphragm (12 only on the right side, and 7 on both sides), three assessed intercostal muscles, and one evaluated abdominal muscles. Twelve included studies used B-mode USG to measure muscle thickness and nine used M-mode USG to measure muscle excursions. Four included studies used SWE to measure muscle stiffness. Two included studies measured strain rate using speckle tracking imaging (STI), and one measured diaphragmatic motion velocity using tissue doppler imaging (TDI)). The examiners’ experiences ranged from experienced (*n* = 13), novice (*n* = 4), to unspecified (*n* = 9).

**Table 1:** Characteristics of included studies

Author	Study design	Participant population	Examiners	Equipment parameters	Target Muscle(s)	Transducer location	Movement	Measurement property
Amerijckx et al. [34], 2020; Belgium	Observational study	Healthy *n* = 67 Male *n* = 31 Female *n* = 36 mean ± SD Age: 22 ± 2 y BMI: 23 ± 3 kg/m2	2 examiners	Esaote MyLab TM one device (Italy);6–13 MHz linear probe; B-mode	Left TrA, IO	Placed transverdally, 2.5 cm medially of the mis-axillary line and halfway between the lowerest rib and ilium	End of natural breathing cycle,end of maximal inspiration, end of maximal expiration	Thickness
Bachasson et al. [23], 2019; France	Observational study	Healthy *n* = 15 Male *n* = 11 mean(range) Age 32(18–43) BMI 24(2.6) Female *n* = 4 age 28(20–44) BMI 21.3(1.3)kg/m2	A trained operator	Aixplorer Ultrasound scanner (V11.2; SuperSonic Imagine, Aixen Provence, France);10- to 2-MHz linear transducer; SWE mode	Right diaphragm	ZOA, on the posterior axillary line vertical to the chest wall at the 8th to 10th intercostal space	NR	Stiffness
Baldwin et al. [35], 2011; Australia	Observational study	Healthy *n *= 13 Male *n* = 6 Female *n* = 7 mean(range) Age 33(20–73) y BMI 25.7 (19.2–30.8) kg/m2	NR	75L38EA with the DP-6600, Shenzhen China; linear array US transducer 10 MHz; B-mode	Right diaphragm	ZOA, against the chest wall at the mid-axillary line of the 9th intercostal space	Expiration to the target volumes of end-expiration	Thickness
Blaney et al. [36], 1998; Australia	Observational study	Healthy *n* = 12 mean(range) Age 18.9(18–22) y	An experienced sonographer	ATL-HDI 3000; M-mode	Diaphragm	NR	Uncoached tidal breathing, upper chest breathing, diaphragmatic breathing, thoracic expansion	Excursion
Boussuges et al. [37], 2009; France	Observational study	Healthy *n* = 210 Male: mean ± SD (range) Age 50 ± 14(20–17) y BMI 23 ± 4 (18–35)kg/m2 Female: Age 49 ± 16(21–77)y BMI 25 ± 5 (16–45)kg/m2	2 examiners	Mylab 30CV; Esaote, Genoa, Italy; 2.5 to 3.5 MHz transducer array; M-mode	Both left and right diaphragm	Right: subcostal area between the midclavicular and anterior axillary lines Left: subcostal area between anterior and mid axillary lines	Quiet breathing, voluntary sniffing, and deep breathing	Excursion
Brown et al. [38], 2018; US	Observational study	Healthy *n* = 45 Female *n* = 31 mean (SD) Age 26.0 (3.4) y BMI 23.4 (2.9) kg/m2	1 novice ultrasonographer, received 8 h of training in ultrasonography	GE Medical Systems, Milwaukee, WI; 8.0 MHz linear array transducer; B-mode	right diaphragm	Zone of apposition, right anterior axillary line and the ninth intercostal space	Peak-inspiration and end-expiration (quiet)	Thickness
Cappellini et al. [39], 2021; Italy	Cross-sectional Study	Healthy *n* = 10 male *n* = 5, female *n* = 5 Male: mean(range) Age: 31(30–32) y BMI: 22.82 (21.65–23.99) kg/m2 Female: Age: 32(29–36) y BMI: 21.54 (19.14–23.94) kg/m2	3 operators, a radiologist, a resident with basic knowledge and skills in ultrasonography, a medical student; all trained for ten sessions on how to recognize the anatomical landmarks used in the protocol proposed	Esaote MyLab 25 System (Esaote, Genoa, Italy); 12 MHz linear probe; B-mode; M-mode	Both left and right diaphragm	Zone of apposition	End-inspiration, end-expiration	Thickness
Dres et al. [22], 2020; France	Cross-sectional Study	Healthy *n* = 23	2 examiners, both experienced in respiratory muscles ultrasound	10–15 MHz linear array transducer; M-mode	Parasternal intercostal muscle	Positioned perpendicular to the anterior thorax surface in the longitudinal scan, at the level of the second right intercostal space, approximately 6–8 cm lateral to the sternal edge with a window visualizing the 2nd and 3rd ribs	End-expiration and at peak inspiration	Thickness
Flattres et al. [26], 2020; France	Cross-sectional Study	Healthy *n* = 15 mean ± SD Age: 26.7 ± 4.6y mean(range) BMI: 22.6 (19.9–26.3) kg/m2	2 examiners, an expert with 4 years of experience in the field of skeletal muscle ultrasound; a novice. Both were trained by the SuperSonic Imagine engineer	SuperSonic Imagine, AixenProvence, France; 4–15 MHz linear transducer; SWE mode	Right diaphragm	Zone of Apposition, at the 8th–10th intercostal space between the right anterior and midaxillary lines	End of expiration	Stiffness
Harper et al. [40], 2013; US	Cross-sectional Study	Healthy *n* = 150 mean ± SD Age 50.6 ± 17.8y BMI 27.9 ± 5.3 kg/m2	2 examiners, trained for several weeks	LOGIQ e; GE Healthcare, Waukesha, WI; 7- to 13- MHz linear array transducer; B-mode	Both left and right diaphragm	Placed transversely over the lowest intercostal space	End of quiet inspiration; end of quiet expiration	Thickness
Marugán et al. [41], 2021; Spain	Cross-sectional Study	Athletes with non-specific lumbopelvic pain *n* = 37, male *n* = 25, female *n* = 12 mean ± SD Age: 31.64 ± 5.56y BMI: 23.14 ± 2.37 kg/m2	2 examiners, more than 4y experience working with the ultrasound technique	Ecube i7; Alpinion Medical System; Seoul, Korea; Linear probe, 8-12 MHz; B-mode	Both left and right diaphragm	Perpendicularly placed with respect to the last intercostal space following the mid-axillary line from the inferior edge of the 11th rib to the superior edge of the 12th rib of the thorax	At maximum inspiration, maximum expiration	thickness
Mohan et al. [42], 2017; Thailand	Observational study	Non-specific low back pain *n* = 9 mean(range) Age 23.33(1.58) y BMI 23.61 (6.31) kg/m2	1 examiner, trained from medical imaging department with 3y of experience	HD 3; Philips Ultrasound, Bothell, USA; 3.5 MHz convex transducer; B-mode	Right diaphragm	Placed over the right subcostal region	NR	Excursion
Nassiri et al. [43], 2019; Iran	Cross-sectional study	Pelvic girdle pain (PGP) *n* = 10 Healthy control *n* = 10 PGP: mean ± SD Age 26.10 ± 5.87y BMI 24.43 ± 2.03 kg/m2 Control: Age 30.90 ± 7.73y BMI 23.48 ± 2.32 kg/m2	1 examiner: an experienced physiotherapist in musculoskeletal ultrasonography	Ultrasonic Scanner, Qsono, China; B-mode with a 7—13 MHz linear array transducer; M-mode: curve transducer	B-mode: both sides diaphragm M-mode: right diaphragm	B-mode: anterior to the anterior axillary line in the intercostal space between the 7th and 8th, or 8th and 9th ribs, at which the diaphragm was more easily visualized M-mode: right mid- clavicular line immediately below the costal margin with firm pressure, and directed medially, cephalad, and dorsally	The end of expiration in quiet breathing; maximal inspiration	Thickness Excursion
Noh et al. [44], 2016; Korea	Observational study	AIS female *n* = 32 Thoracic curve: *n* = 17 mean ± SD Age 14.1 ± 1.9 Cobb angle 29.5 ± 17.0y Thoracolumbar curve: *n* = 15 Age 14.3 ± 1.8y Cobb angle 20.7 ± 7.9	2 examiners	SONOACE X4, Medison, Seoul, Korea; 3.5 MHz curvilinear transducer; M-mode	Both left and right diaphragm	Sub-costal spaces between the midclavicular and anterior axillary lines (right); Sub-costal spaces between the anterior and mid axillary lines (left)	At the end of inspiration and expiration during tidal breathing	Excursion
Noh et al. [45], 2014; Korea	Observational study	Healthy *n* = 14 male *n* = 9 female *n* = 5 mean ± SD Age 28.4 ± 3.0y	NR	SONOACE 6000, Medison, Seoul, Korea; 3.5 MHz sector transducer; M-mode	Right diaphragm	Right sub-costal margin between the midclavicular and anterior axillary lines	At the end of inspiration and expiration during tidal breathing	Excursion
Oppersma et al. [46], 2017; Netherlands	Observational study	Healthy *n* = 15 male *n* = 7 mean(range) Age 21.3 (2.3) y BMI 21.6(1.7) kg/m2	NR	Vivid E 9TM ultrasound machine (General Electric Healthcare, Horton, Norway); 9-MHz linear transducer; Speckle tracking	Right diaphragm	Right anterior axillary line longitudinal to the body axis (between the 9th-11th intercostal space)	At end expiration, end inspiration	Strain
Orde, et al. [47], 2016; US	Observational study	Healthy *n* = 50 female *n* = 28 mean(range) Age 37(30.2–39.8) y BMI 22.8 (20.4–24.9) kg/m2	2 examiners: Australian Intensive Care specialist, board certified in standard and advanced echocardiography in America	Vivid E9, General Electric Healthcare, Milwaukee, WI); linear array transducer (2.5–8 MHz) and a phased array transducer (1.6–6 MHz); M-mode; Speckle tracking	Right diaphragm	Thickness & strain: right anterior axillary line at approximately the ninth intercostal space Excision: subcostally on the right mid-clavicular line	From the end of expiration through the end of inspiration	Thickness Excursion Strain
Pietton et al. [25], 2021; France	Cross-sectional Study	Healthy: *n* = 19 mean ± SD Age: 12.6 ± 1.7y BMI: 19.3 ± 2 kg/m2 14 girls and five boys AIS: *n* = 16 Age: 13 ± 2.5 y BMI: 17.9 ± 1.6 kg/m2 15 girls and one boy	3 examiners: 2y, 6 m, 2 m experience of ultrasound measurements	Aixplorer (Supersonic Imagine, Aixenprovence, France); Linear; SWE mode	Right intercostal muscle	T5-T6 right intercostal space, at the mid-axillary line	During normal breathing and in apnea. Apnea was performed at tidal volume	Stiffness
Scarlata et al. [48], 2019; Italy	Cross-sectional Study	Healthy *n* = 66 Male *n* = 30 Female *n* = 36 mean (SD) Age: 40 (15)y BMI: 24.2 (3.5) kg/m2	2 examiners	Exagyne—Echo Control Medical- ECM, Angoulme, France; linear probe; B-mode, M-mode	Right diaphragm	Placed on the line between the eighth and ninth intercostal spaces, midway between the antero- and mid-axillary lines	End of deep inspiration, end of normal expiration	Thickness
Scarlata et al. [49], 2018; Italy	Observational study	Healthy *n* = 100 Male *n* = 49 mean (SD) Age 40 (15)y BMI 24.4 (3.8) kg/m2	3 examiners: experienced	ECM [Echo Control Medical] in Angouleme, France; convex probe and frequencies between 2.5 and 3.5 MHz; M-mode	Right diaphragm	Placed subcostal, right and anterior to the mid-clavicular line	Quiet and deep breathing	Excursion
Soilemezi et al. [50], 2020; Greece	Cross-sectional Study	Healthy: *n* = 20 male *n* = 10 female *n* = 10 Age range 25-48y	2 examiners	Philips Sparq ultrasound machine; phased array 2–4 MHz probe; Tissue Doppler imaging (TDI)	Right diaphragm	Placed in the subcostal position between the midclavicular and anterior axillary lines	Breathing spontaneously	Diaphragmatic motion velocity
Wallbridge et al. [51], 2018; Australia	Observational study	Stable COPD *n* = 20 Male *n* = 16 Female *n* = 4 mean(range) Age 71.5 (62.3–78.8) y BMI 23.5 (20.9–30) kg/m2	An examiner: with 8 years of ultrasound experience and qualifications in respiratory ultrasound. Images were reviewed by a second reader with respiratory ultrasound experience to assess inter-rater reliability	Shenzhen Mindray Bio-Medical Electronics Co. Ltd. Shenzen, China; 6–14 MHz linear; B-mode	Bilateral intercostal muscles	2nd and 3rd parasternal intercostal muscles bilaterally	End-tidal inspiration	Thickness
Xu et al. [24], 2021; China	Cross-sectional Study	Stable COPD: *n* = 43 mean ± SD Age: 64.5 ± 7.9y BMI: 22.6 ± 3.3 kg/m2 Control: *n* = 34 Age: 63.8 ± 7y BMI: 24 ± 2.8 kg/m2	1 examiner: 3y experience and was thoroughly trained in using SWE on the diaphragm	Logiq E9 (GE Healthcare, Wauwatosa, WI, USA) ultrasound system; 9 MHz linear transducer; SWE mode	Right diaphragm	Zone of apposition, between the right anterior and midaxillary lines vertical to the chest wall at the 8th to10th intercostal space	End of expiration	Stiffness
Ziaeifar et al. [52], 2021; Iran	Case–control study	LBP *n* = 37 mean ± SD Age 38.29 ± 10.95y BMI 24.65 ± 3.01 kg/m2 Healthy: *n* = 34 Age 32.82 ± 10.43y BMI 23.38 ± 3.48 kg/m2	An experienced and expert radiologist	Toshiba, Aplio 300, Tokyo, Japan; Excursion: 3.5 MHz curvilinear transducer; M-mode. Thickness: 7.5 MHz linear array transducer; B-mode	Both left and right diaphragm	Excursion: the lower intercostal area between the midclavicular and anterior axillary lines for the right diaphragm and between the anterior and midaxillary lines for the left side. Thickness: zone of apposition, between the mid and anterior axillary lines on the right and left sides, typically between the 8th and 10th intercostal spaces diaphragm with the transducer spanning two ribs	Quiet breath; Deep breath	Excursion Thickness

*Abbreviations*: *BMI* body mass index, *SD* standard deviation, *NR* not reported, *TrA* Transverse abdominals, *IO* internus obliquus, *LBP* low back pain, *AIS* adolescent idiopathic scoliosis, *COPD* chronic obstructive pulmonary disease

### Ultrasound measurement approach

Eight included articles measured the thickness [35, 38–41, 43, 48, 52], nine measured excursion [36, 37, 42–45, 47, 49, 52], three measured stiffness [23, 24, 26], two measured strain [46, 47], and one measured motion velocity of diaphragm [50]; one included study measured the thickness [51], and one measured stiffness of intercostal muscles [25]; one included study measured the thickness of transverse abdominals and internus obliquus with different approaches [34]. The details of each measurement approach are described in Supplementary File-S 4.

### Reliability

**Fig. 2:**
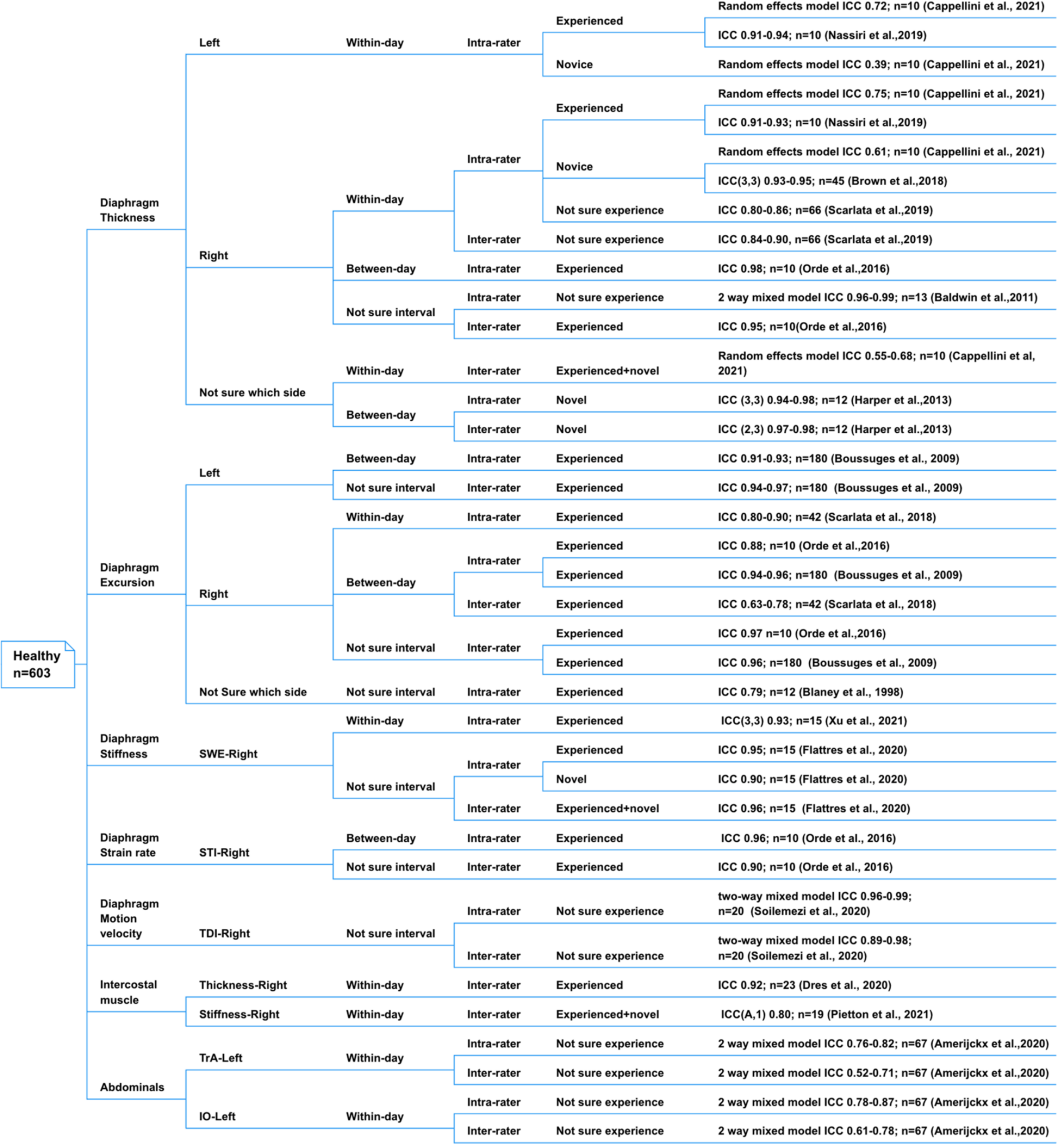
Reliability of healthy population

**Fig. 3:**
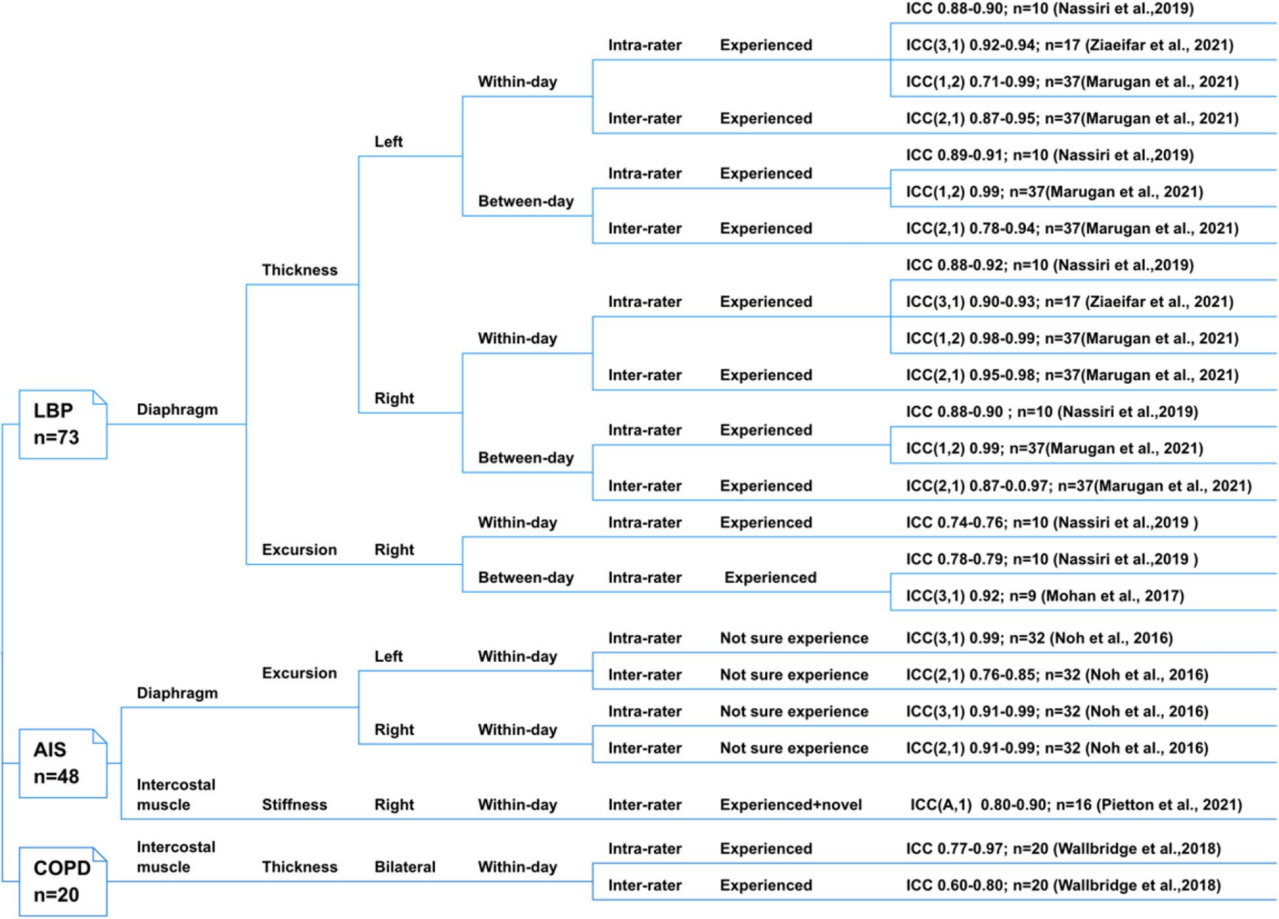
Reliability of LBP, AIS, and COPD population

Figures 2 and 3 illustrate the reliability of using different types of USG to measure various respiratory muscle characteristics in different populations. Tables 2 and Supplementary File-S 5 show the COSMIN scores and the rating of each study, as well as the quality of evidence regarding the reliability of each type of respiratory muscle measurement based on all included studies and separated populations, respectively. Overall, moderate-quality evidence supported sufficient within-day intra-rater reliability measuring right diaphragm thickness and sufficient between-day intra-rater reliability measuring right diaphragm excursion with B-mode and M-mode USG. The quality of evidence for the measurement properties was low or very low.

**Table 2:** Quality assessments and level of evidence based on all included studies – reliability

Targeted Muscle	Measurement Property	Study	Operator Experience	Position	Sample Size	Methodological quality (COSMIN)	Results rating						Evidence level
							**Within-day**		**Between-day**		**Not sure interval**		
							**Intra-rater**	**Inter-rater**	**Intra-rater**	**Inter-rater**	**Intra-rater**	**Inter-rater**	
Diaphragm-Left	Thickness	*Nassiri *et al*., 2019*	Experienced	Hook lying, pillow under knees, arms on sides	10	Doubtful	+						Within-day intra-rater low ( +)
		*Nassiri *et al*., 2019*	Experienced	Hook lying, pillow under knees, arms on sides	10	Doubtful	+		+				
		*Cappellini *et al*., 2021*	Experienced	semi-recumbent position, head up45	10	Adequate	+						
		*Ziaeifar *et al*., 2021*	Experienced	spine, knees bend 30 degree, arms cross over the chest	17	Adequate	+						
		*Marugan *et al*., 2021*	Experienced	supine without pillow	37	Very good	+	+	+	+			
		*Cappellini *et al*., 2021*	Novice	semi-recumbent position, head up45	10	Adequate	-						
Diaphragm-Right	Thickness	*Nassiri *et al*., 2019*	Experienced	Hook lying, pillow under knees, arms on sides	10	Doubtful	+						Within-day intra-rater moderate, ( +)
		*Nassiri *et al*., 2019*	Experienced	Hook lying, pillow under knees, arms on sides	10	Doubtful	+		+				
		*Cappellini *et al*., 2021*	Experienced	semi-recumbent position, head up45	10	Adequate	+						
		*Orde *et al*., 2016*	Experienced	semi-recumbent position, head up45	10	Doubtful			+			+	
		*Ziaeifar *et al*., 2021*	Experienced	spine, knees bend 30 degree	17	Adequate	+						
		*Marugan *et al*., 2021*	Experienced	supine	37	Very good	+	+	+	+			
		*Cappellini *et al*., 2021*	Novice	semi-recumbent position, head up45	10	Adequate	-						
		*Brown *et al*., 2018*	Novice	supine, sitting, standing	45	Doubtful	+						
		*Baldwin *et al*., 2011*	Unspecified	semi-recumbent position	10	Doubtful					+		
		*Scarlata *et al*., 2019*	Unspecified	recumbent position	66	Inadequate	+	+					
Diaphragm-Left	Excursion	*Boussuges *et al*., 2009*	Experienced	standing	180	Doubtful			+			+	Low, ( +)
		*Noh *et al*., 2016*	Experienced	supine	32	Doubtful	+	+					Very low, ( +)
Diaphragm-Right	Excursion	*Scarlata *et al*., 2018*	Experienced	spine	42	Doubtful	+			±			Between-day intra-rater: moderate, ( +)
		*Orde *et al*., 2016*	Experienced	semi-recumbent position, head up45	10	Doubtful			+			+	
		*Boussuges *et al*., 2009*	Experienced	standing	180	Doubtful			+			+	
		*Nassiri *et al*., 2019*	Experienced	Hook lying, pillow under knees, arms on sides	10	Doubtful	+		+				
		*Mohan *et al*., 2017*	Experienced	head elevated to 30 degree	9	Doubtful			+				
		*Noh *et al*., 2016*	Experienced	supine	32	Doubtful	+	+					
Diaphragm-Right	Stiffness	*Xu *et al*., 2021*	Experienced	supine	15	Very good	+						Low, ( +)
		*Flattres *et al*., 2020*	Experienced	supine	15	Doubtful					+		Very low, ( +)
		*Flattres *et al*., 2020*	Novice	supine	15	Doubtful					+		Very low, ( +)
		*Flattres *et al*., 2020*	Experienced + novice	supine	15	Doubtful						+	Very low, ( +)
	Strain rate	*Orde *et al*., 2016*	Experienced	semi-recumbent position, head up45	10	Doubtful			+			+	Very low, ( +)
	Motion velocity	*Soilemezi *et al*., 2020*	Experienced	supine with back elevated at 30 degree	20	Doubtful					+	+	Very low, ( +)
Intercostal muscle	Thickness	*Dres *et al*., 2020*	Experienced	supine	23	Doubtful		+					Very low, ( +)
		*Wallbridge *et al*., 2018*	Experienced	supine	20	Doubtful	+	±					Very low, ( +)
	Stiffness	*Pietton *et al*., 2021*	Experienced	supine	16	Doubtful		+					Very low, ( +)
		*Pietton *et al*., 2021*	Experienced	supine	16	Doubtful		+					Very low, ( +)
TrA-Left	Thickness	*Amerijckx *et al*. 2020*	Experienced	standing	67	Doubtful	+	±					Very low, ( +)
IO-Left	Thickness	*Amerijckx *et al*. 2020*	Experienced	standing	67	Doubtful	+	±					Very low, ( +)

*Abbreviations*: *TrA* Transverse abdominals, *IO* internus obliquus, *Not sure* not sure time interval

### Findings for different populations

#### Healthy individuals

##### Diaphragm thickness: intra- and inter-rater reliability

Seven included studies [35, 38–40, 43, 47, 48] examined the reliability of using B-mode USG for diaphragm thickness measurements in healthy individuals. Two included studies reported sufficient within-day intra-rater reliability of measuring left hemidiaphragm thickness by experienced operators (ICC = 0.72–0.94) [39, 43] but insufficient within-day intra-rater reliability for novice operators (ICC = 0.39) [39]. Likewise, two included studies reported sufficient within-day intra-rater reliability of measuring right hemidiaphragm thickness by experienced operators (ICC = 0.75–0.93) [39, 43] or an operator of unknown experience (ICC = 0.84–0.90) [48], but insufficient within-day intra-rater reliability by novice operators (ICC = 0.61–0.95) [38, 39]. One included study reported sufficient between-day intra-rater reliability for an experienced operator to measure right hemidiaphragm thickness (ICC = 0.98) [47]. Two included studies [47, 48] reported sufficient within-day inter-rater reliability (ICC = 0.84–0.90) [48] of measuring right hemidiaphragm thickness by an operator of unknown experience, and sufficient inter-rater reliability (ICC = 0.95) [47] for experienced operators with unknown years of experience. Two included studies [39, 40] reported sufficient between-day intra- and inter-rater reliability (ICC = 0.94–0.98; 0.97–0.98) [40] and insufficient inter-rater (between an experienced and a novice operator) reliability (ICC = 0.55–0.68), although the measurement side was unspecified [39].

Four [35, 38, 43, 47] out of seven (57%) included studies were rated as doubtful for the methodological quality of measuring diaphragm thickness. Two [39, 40] were rated as adequate and one was rated as inadequate [48]. Collectively, the quality of evidence for the intra- and inter-rater reliability of using B-mode USG to measure diaphragm thickness was very low.

##### Diaphragm excursion: intra- and inter-rater reliability

Four included studies [36, 37, 47, 49] reported the reliability of using M-mode USG to measure left [37] and right [36, 37, 47, 49] diaphragm excursion by experienced operators, but the methodological quality of these studies was doubtful. The between-day intra-rater reliability was consistently reported as sufficient on both sides (ICC = 0.80–0.96) [37, 47]. However, the between-day inter-rater reliability was inconsistent (ICC = 0.63–0.78) because insufficient reliability (ICC = 0.63) was reported when measuring the diaphragm excursion during quiet breathing [49]. One included study reported intra-rater reliability without stating the side of the hemidiaphragm and the measurement interval (ICC = 0.79) [36]. There was low-quality evidence that the between-day intra-rater reliability of M-mode USG in measuring bilateral diaphragmatic excursion was sufficient. The evidence for other excursion measurements was very low.

##### Diaphragm stiffness: intra- and inter-rater reliability

Two included studies [24, 26] reported the reliability of using SWE to measure right diaphragmatic stiffness. The within-day intra-rater reliability was sufficient (ICC = 0.93) [24], and the COSMIN rating was very good. One included study reported sufficient inter-rater reliability (ICC = 0.96) without specifying whether it was within- or between-day measurements [26]. There was low-quality evidence that the within-day intra-rater reliability of SWE in measuring right diaphragmatic stiffness was sufficient.

##### Diaphragm strain rate and motion velocity: intra- and inter-rater reliability

One included study [47] reported sufficient between-day intra-rater reliability of using STI to measure diaphragmatic strain rate (ICC = 0.96), but COSMIN rating was doubtful, and the evidence was very low. One included study [50] which used TDI to measure diaphragmatic motion velocity without specifying the time interval, and reported sufficient reliability of intra and inter-rater reliability (ICC = 0.96–0.99; ICC = 0.89–0.98), but its COSMIN rating was doubtful and evidence was very low.

##### Intercostal muscle thickness and stiffness: within-day inter-rater reliability

One included study measured intercostal muscle thickness [22] and one measured intercostal muscle stiffness [25]. Both studies reported sufficient within-day inter-rater reliability (ICC = 0.92; ICC = 0.80). The COSMIN ratings of both studies were doubtful, and the evidence was very low.

##### Abdominal muscle thickness: within-day intra- and inter-rater reliability

One included study [34] reported sufficient within-day intra-rater reliability (ICC = 0.76–0.82; ICC = 0.78–0.87) but inconsistent within-day inter-rater reliability (ICC = 0.52–0.71; ICC = 0.61–0.78) for using B-mode USG to quantify left transverse abdominals and internal obliquus thickness during different breathing phases. All the evidence was very low.

#### LBP

##### Diaphragm thickness: intra- and inter-rater reliability

Three included studies [41, 43, 52] reported sufficient intra- and inter-rater reliability of using B-mode USG for bilateral diaphragmatic thickness measurements by experienced operators (left: ICC = 0.71–0.99; right: ICC = 0.87–0.99). The COSMIN ratings of the three studies were doubtful [43], adequate [52], and very good [41]. Overall, low-quality evidence supported that the within- and between-day intra- and inter-rater reliability of B-mode USG in measuring bilateral diaphragmatic thickness in supine lying individuals with LBP was sufficient.

##### Diaphragm-excursion: intra- and inter-rater reliability

Two included studies [42, 43] consistently reported sufficient within- (ICC = 0.74–0.76) and between-day intra-rater reliability (ICC = 0.78–0.92) of using M-mode USG to measure the right hemidiaphragm excursion. The COSMIN ratings of both studies were doubtful, and the quality of evidence was very low.

#### AIS

##### Diaphragm-excursion and intercostal muscles-stiffness: intra- and inter-rater reliability

Two included studies reported sufficient within-day intra- and inter-rater reliability of using M-mode USG to measure bilateral diaphragmatic excursion (ICC = 0.76–0.99) [44] and using SWE to measure right intercostal muscle stiffness (ICC = 0.80–0.90) [25] in teenagers with AIS. The COMSIN ratings of both studies were doubtful, and the relevant evidence was very low.

#### COPD

##### Intercostal muscle thickness: intra- and inter-rater reliability

One included study [51] reported sufficient within-day intra-rater reliability (ICC = 0.77–0.97) and inconsistent within-day inter-rater reliability (ICC = 0.60–0.80) for measuring intercostal muscle thickness using B-mode USG. The insufficient result was reported in measuring intercostal muscle thickness at right second and third intercostal levels. The COSMIN rating of this study was doubtful, and the evidence was very low.

### Validity

**Fig. 4:**
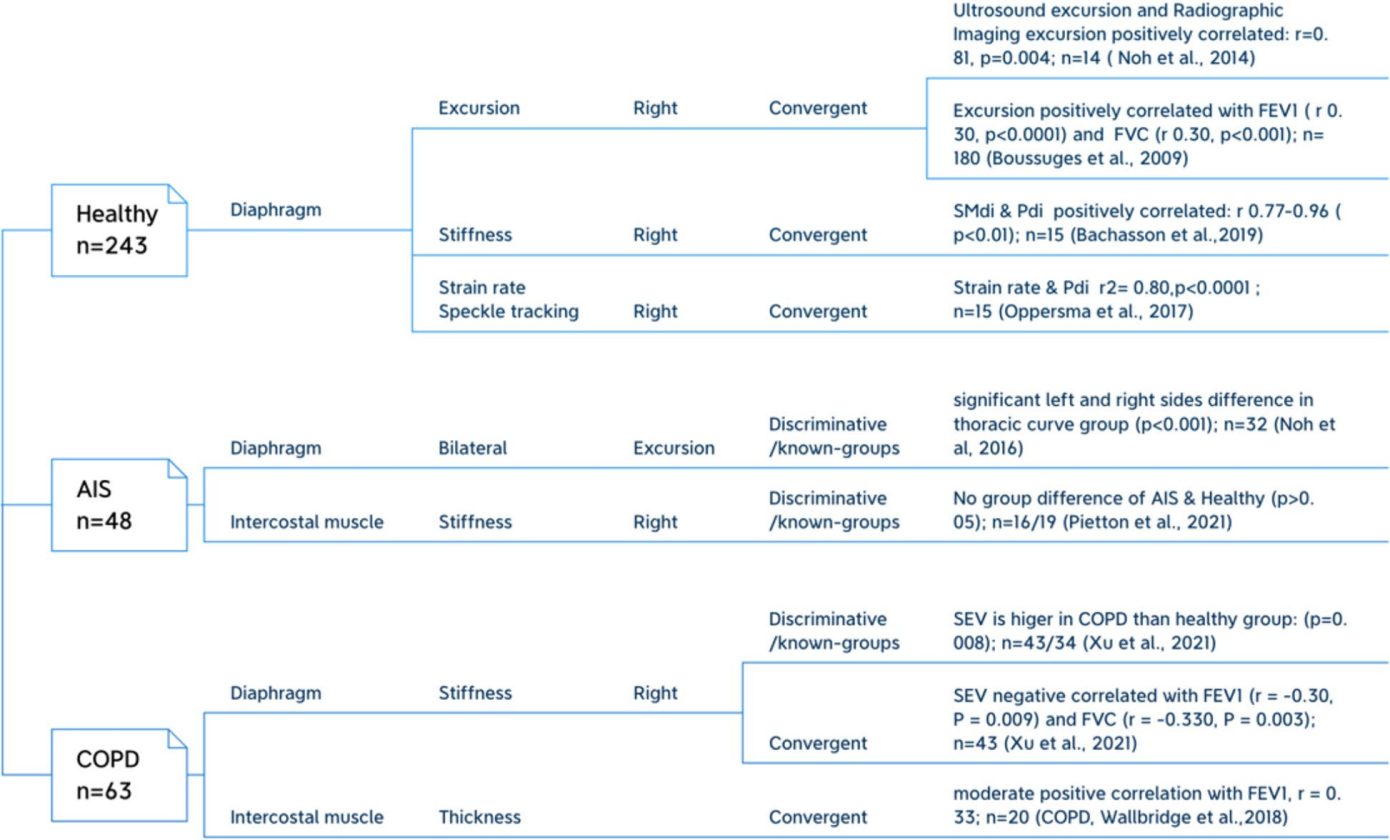
Validity of included studies

The validity of relevant included studies, the methodological quality assessment of each study, and the relevant evidence are shown in Fig. 4 and Table 3.

**Table 3:** Quality assessments and level of evidence – validity

Polupation	Targeted Muscle	Convergent	Discriminative/known-groups	Study	Sample Size	Quality rating(COSMIN)	Results rating	Level of Evidence
Healthy	Diaphragm-Right	Excursion & Radiographic Image		*Noh *et al*., 2014*	14	Very good	+	*n* = 14, low ( +)
		Excursion & FEV1, FVC		*Boussuges *et al*., 2009*	180	Very good	+	*n* = 180, high ( +)
		Stiffness & Pdi		*Bachasson *et al*., 2019*	15	Very good	+	*n* = 15, low ( +)
		Strain rate & Pdi		*Oppersma *et al*., 2017*	15	Very good	+	*n* = 15, low ( +)
AIS	Diaphragm-Bilateral		Excursion: AIS Left & right side	*Noh *et al*., 2016*	32/32	Adequate	+	*n* = 32, very low ( +)
	Intercostal muscle-Right		Stiffness: AIS & Healthy	*Pietton *et al*., 2021*	16/19	Doubtful	-	*n* = 16, very low (-)
COPD	Diaphragm-Right	Stiffness & FEV1,FVC		*Xu *et al*., 2021*	43	Very good	+	*n* = 43, low ( +)
			Stiffness: COPD & Healthy	*Xu *et al*., 2021*	43/34	Doubtful	+	*n* = 43, very low ( +)
	Intercostal muscle	Thickness & FEV1		*Wallbridge *et al*., 2018*	20	Adequate	+	*n* = 20, very low ( +)

#### Healthy

##### Convergent validity

Four studies [23, 37, 45, 46] involving 243 healthy participants reported different convergent validity of using different types of USG to evaluate diaphragmatic morphometry or functions. Positive correlations were noted between the diaphragmatic excursion as measured by M-mode USG and radiographic imaging (X-ray) [45], between the diaphragmatic excursion and forced expiratory volume in the first second (FEV_1_) or forced vital capacity (FVC) [37], as well as between diaphragmatic stiffness [23] or strain rate [46] and transdiaphragmatic pressure (Pdi). The methodological quality of these included studies was very good, and the validity values were rated as sufficient. There was high-quality evidence to support the convergent validity between diaphragmatic excursion and FEV_1_ or FVC, [37] while all others were low.

#### AIS

##### Discriminative/known-groups validity

One included study reported a significant difference in the left and right side diaphragmatic excursion among participants with a thoracic curve, with an adequate COSMIN rating [44]. Another included study reported no significant group difference in the stiffness of intercostal muscles between participants with and without AIS [25], but its COSMIN rating was doubtful. The quality of evidence for both conditions was low.

#### COPD

##### Convergent and discriminative/known-groups validity

One included study [24] reported both discriminative/known-groups and convergent validity of diagrammatic stiffness measurement. Shear wave velocity in the COPD group was significantly higher than that of the healthy controls, and shear wave velocity was negatively correlated with FEV_1_ or FVC. Another included study [51] revealed a significant positive correlation between intercostal muscle thickness and FEV_1_. All these results were rated as sufficient. The methodological quality of these two studies on convergent validity was rated as doubtful or adequate, and the overall quality of evidence was very low. While the methodological quality and quality of evidence of the first study [24] on discriminative/known-groups validity was rated as very good and low, respectively.

## Discussion

Our review found moderate-quality evidence to support sufficient within-day intra-rater reliability of B-mode USG in measuring right diaphragmatic thickness among people with LBP, sufficient between-day intra-rater reliability of M-mode USG in measuring right diaphragmatic excursion in non-hospitalized individuals. The quality of evidence for all other measurements in relevant included studies and separated populations was low or very low. High-quality evidence supported the positive correlation between diaphragmatic excursion and FEV_1_ or FVC in healthy individuals [37]. However, the quality of evidence for the validity between various USG measurement parameters and other comparators was low or very low.

There are several possible reasons for the observed low quality of evidence. According to the grading criteria, most of the included studies were downgraded by their small sample sizes and poor methodological quality. Because the included studies were heterogeneous in terms of the position of participants, breathing phases during measurements, the definition of operators' experiences, and the types of ICC model, no meta-analysis was conducted. Therefore, the sample size for each measurement parameter in each condition was very small. Additionally, most included studies were rated with doubtful methodological quality according to the latest version of the ClinROMs checklist [31]. Unlike the COSMIN PROMs checklist (box 6 for reliability assessment) [32], the ClinROMs checklist was developed for clinician‐reported outcome measures, which include readings from imaging modalities and ratings based on observations such as USG. Studies involving ClinROMs were more complicated because the involved patients, clinicians, and devices might affect the methodological quality. Therefore, the ClinROMs checklist adds items related to these factors. Most of our included studies lost scores on items 4 and 5 (related to professionals). Four included studies published in 2021 developed their study design based on the ClinROMs checklists. Therefore, they were rated as very good or adequate [24, 39, 52, 53]. Collectively, earlier research that followed the previous COSMIN checklist in designing their studies yielded low methodological quality.

As expected, the intra-rater reliability was higher than the inter-rater reliability, and the reliability of experienced operators was higher than novice operators. The relatively lower inter-rater reliability in the current review concurs with previous findings on critically ill patients [19]. Novice operators have low reliability in performing USG measurements of muscles because USG is operator dependent. Specifically, the placement of a probe at the target location (zone of apposition, subcostal, intercostal) and the selection of the best image on each measurement highly depends on the operator’s experience. Such measurements are even more challenging for dynamic diaphragm measurements.

Although no meta-analysis was conducted, the reliability of measuring right hemidiaphragm seems to be higher than that of the left side. Using any type of USG to investigate diaphragm needs adjacent structures to provide a good acoustic window. Liver provides a good acoustic window for the right hemidiaphragm investigation, whereas the measure on the left hemidiaphragm is more challenging for novice operators given the smaller spleen window and the interference of gas in the gastrointestinal tract [37, 54, 55].

The comparators in convergent validity studies included Pdi, FEV_1_, and FVC. Pdi is a golden standard for evaluating diaphragm function but it is invasive [19]. The strong positive correlation between Pdi and diaphragmatic stiffness [23] or strain rate [46] suggest that SWE and STI may noninvasively assess diaphragm functions. FEV_1_ and FVC are commonly used to quantify respiratory function of patients with COPD [56]. The sufficient correlations between FEV_1_ or FVC and respiratory muscle stiffness [24], thickness [51] and excursion [37] suggest that the SWE, B-mode and M-mode USG can be used to assess respiratory functions. Further studies should explore the measurement properties of other non-invasive measurements of respiratory muscle properties (e.g., magnetic resonance imaging).

The known-groups validity studies found that certain USG assessments of diaphragm parameters could be used to discriminate people with and without diseases [24, 25, 44]. Notably, the reported discriminative/known-groups validity in patients with AIS suggested that M-mode USG-measured diaphragmatic excursion might help differentiate the bilateral hemi-diaphragmatic function in patients with different severity of the thoracic curve [44]. However, the intercostal muscle stiffness cannot differentiate people with and without AIS [25], a study on patients with COPD suggests that SWE-measured diaphragmatic stiffness can differentiate people with and without COPD [24].

The evidence regarding SWE, STI, and TDI was low summarized because of the limited number of included studies. SWE generates shear waves that propagate through tissues in the transverse plane causing shear displacements, which can be tracked to calculate shear wave velocity or shear modulus [27]. Shear wave velocity is faster in stiffer tissues, but decreases significantly with the thickness in thin tissues, especially when the thickness is less than 1.5 cm [57, 58]. Therefore, shear wave velocity is affected by muscle mechanical properties and thickness in very thin tissues. Because both diaphragm and intercostal muscles are thin (0.13–0.76 cm) [59], the validity of using SWE to measure inspiratory muscle stiffness should be interpreted with caution [29]. Further, the limited penetration depth, high sensitivity to sensor pressure and angle, and the dependence of shear modulus on the probe orientation are the disadvantages of SWE [27, 60]. Future studies should take muscle thickness into consideration if SWE is used to measure respiratory muscle stiffness.

Both STI and TDI are strain rate imaging, which measure the differences in motion and velocity within tissues. They are commonly used in echocardiographic imaging to assess regional myocardial function [61, 62]. Speckles are small groups of tissue pixels with specific grayscale characteristics created by the interaction of ultrasound beams and tissues and can be used to calculate the tissue strain and strain rate [62]. STI technique identifies and tracks the same speckle throughout the movement cycle. While TDI measures the longitudinal strain and strain rate (one dimension, ultrasound beam should be parallel to the direction of tissue motion), STI is independent of the angle and beam directions, and allows the tracking in two dimensions [63]. Therefore, STI is better than TDI in investigating the motion of diaphragm which may better reflect diaphragmatic contractibility. More studies are warranted to use these two novel techniques to investigate respiratory muscles.

### Limitations

The current review had several limitations. First, the included studies were heterogenous, which precluded meta-analysis. Second, the use of the updated and stricter ClinROMs checklist led to the downgrade of the quality of evidence, although it was essential. Third, no included studies evaluated the responsiveness of various USG measurements, which may limit its clinical usage.

## Conclusions

This is the first systematic review on the evidence regarding the measurement properties of using various types of USG to evaluate respiratory muscle characteristics in non-hospitalized populations. Although separate included studies revealed sufficient reliability and validity of using these USG technologies to assess the morphometry, function, and mechanical properties of respiratory muscles in non-hospitalized individuals, the respective quality of evidence was low due to the limited number of relevant studies. More high-quality large-scale studies are warranted to establish the reliability and validity of using various types of USG assessments to measure different respiratory muscle characteristics in different populations.

### Supplementary Information

**Additional file 1.**

## Data Availability

The data that support the findings of this study are available from the corresponding author upon reasonable request.

## Abbreviations

AIS: Adolescent idiopathic scoliosis
B-mode: Brightness-mode
ClinROMs: Clinician‐Reported Outcome Measures
CI: Confidence interval
COPD: Chronic obstructive pulmonary disease
COSMIN: The Consensus-based Standards for the Selection of Health Measurement Instruments
FEV1: Forced expiratory volume in the first second
FVC: Forced vital capacity
ICC: Intra-class correlation coefficient
LBP: Low back pain
M-mode: Motion-mode
Pdi: Transdiaphragmatic pressure
PROMs: Patient‐Reported Outcome Measures
STI: Speckle tracking imaging
SWE: Shear wave elastography
TDI: Tissue doppler imaging
USG: Ultrasonography

## References

[1] Bains, K.N.S., S. Kashyap, and S.L. Lappin, Anatomy, thorax, diaphragm, in StatPearls. 2021, StatPearls Publishing. 30137842

[2] Meilleur, K.G., M.M. Linton, J. Fontana, A. Rutkowski, J. Elliott, M. Barton, et al., Comparison of sitting and supine forced vital capacity in collagen VI-related dystrophy and laminin α2-related dystrophy. 2017(1099–0496 (Electronic)). 10.1002/ppul.23622 PMC6309368 28085238

[3] Nason LK, Walker CM, McNeeley MF, Burivong W, Fligner CL, Godwin JD (2012). Imaging of the diaphragm: anatomy and function. Radiographics.

[4] Smith MD, Russell A, Hodges PW (2014). The relationship between incontinence, breathing disorders, gastrointestinal symptoms, and back pain in women: a longitudinal cohort study. Clin J Pain.

[5] Smith MD, Russell A, Hodges PW (2009). Do incontinence, breathing difficulties, and gastrointestinal symptoms increase the risk of future back pain?. J Pain.

[6] Kolar P, Sulc J, Kyncl M, Sanda J, Cakrt O, Andel R (2012). Postural function of the diaphragm in persons with and without chronic low back pain. J Orthop Sports Phys Ther.

[7] Janssens L, Brumagne S, McConnell AK, Raymaekers J, Goossens N, Gayan-Ramirez G (2013). The assessment of inspiratory muscle fatigue in healthy individuals: a systematic review. Respir Med.

[8] Boushel R (2010). Muscle metaboreflex control of the circulation during exercise. Acta Physiol (Oxf).

[9] Tiller NB (2019). Pulmonary and respiratory muscle function in response to marathon and ultra-marathon running: a review. Sports Med.

[10] De Troyer A, Boriek AM (2011). Mechanics of the respiratory muscles. Compr Physiol.

[11] Sieck GC, Ferreira LF, Reid MB, Mantilla CB (2013). Mechanical properties of respiratory muscles. Compr Physiol.

[12] Shi ZH, Jonkman A, de Vries H, Jansen D, Ottenheijm C, Girbes A (2019). Expiratory muscle dysfunction in critically ill patients: towards improved understanding. Intensive Care Med.

[13] Mercurio G, D'Arrigo S, Moroni R, Grieco DL, Menga LS, Romano A (2021). Diaphragm thickening fraction predicts noninvasive ventilation outcome: a preliminary physiological study. Crit Care.

[14] Goligher, E.C., M.E. Laghi F Fau - Detsky, P. Detsky Me Fau - Farias, A. Farias P Fau - Murray, D. Murray A Fau - Brace, L.J. Brace D Fau - Brochard, et al., Measuring diaphragm thickness with ultrasound in mechanically ventilated patients: feasibility, reproducibility and validity. Reliability of bedside ultrasound of limb and diaphragm muscle thickness in critically ill children. 2015(1432–1238 (Electronic)).

[15] Umbrello M, Formenti P, Longhi D, Galimberti A, Piva I, Pezzi A (2015). Diaphragm ultrasound as indicator of respiratory effort in critically ill patients undergoing assisted mechanical ventilation: a pilot clinical study. Crit Care.

[16] Dubé BP, Dres M, Mayaux J, Demiri S, Similowski T, Demoule A (2017). Ultrasound evaluation of diaphragm function in mechanically ventilated patients: comparison to phrenic stimulation and prognostic implications. Thorax.

[17] Demoule A, Dubé BP, Mayaux J, Demiri S, Similowski T, Dres M (2017). Validation of ultrasound to assess diaphragm function in mechanically ventilated patients. Ann Intensive Care.

[18] Nascimento TS, de Queiroz RS, Ramos ACC, Martinez BP, Da Silva ESCM, Gomes-Neto M (2021). Ultrasound protocols to assess skeletal and diaphragmatic muscle in people who are critically ill: a systematic review. Ultrasound Med Biol.

[19] Zambon M, Greco M, Bocchino S, Cabrini L, Beccaria PF, Zangrillo A (2017). Assessment of diaphragmatic dysfunction in the critically ill patient with ultrasound: a systematic review. Intensive Care Med.

[20] Formenti P, Umbrello M, Dres M, Chiumello D (2020). Ultrasonographic assessment of parasternal intercostal muscles during mechanical ventilation. Ann Intensive Care.

[21] Wallbridge, P., S. Parry, S. Das, C. Law, G. Hammerschlag, L. Irving, et al., Parasternal intercostal muscle ultrasound measurements correlate with disease severity in chronic obstructive pulmonary disease: A pilot study. European Respiratory Journal, 2018. 52. 10.1038/s41598-018-33666-7 PMC6189142 30323179

[22] Dres M, Dubé B-P, Goligher E, Vorona S, Demiri S, Morawiec E (2020). Usefulness of parasternal intercostal muscle ultrasound during weaning from mechanical ventilation. Anesthesiology.

[23] Bachasson D, Dres M, Niérat MC, Gennisson JL, Hogrel JY, Doorduin J (2019). Diaphragm shear modulus reflects transdiaphragmatic pressure during isovolumetric inspiratory efforts and ventilation against inspiratory loading. J Appl Physiol (1985).

[24] Xu J-H, Wu Z-Z, Tao F-Y, Zhu S-T, Chen S-P, Cai C (2021). Ultrasound shear wave elastography for evaluation of diaphragm stiffness in patients with Stable COPD: a pilot trial. J Ultrasound Med.

[25] Pietton R, David M, Hisaund A, Langlais T, Skalli W, Vialle R (2021). Biomechanical evaluation of intercostal muscles in healthy children and adolescent idiopathic scoliosis: a preliminary study. Ultrasound Med Biol.

[26] Flattres A, Aarab Y, Nougaret S, Garnier F, Larcher R, Amalric M (2020). Real-time shear wave ultrasound elastography: a new tool for the evaluation of diaphragm and limb muscle stiffness in critically ill patients. Crit Care.

[27] Taljanovic MS, Gimber LH, Becker GW, Latt LD, Klauser AS, Melville DM (2017). Shear-wave elastography: basic physics and musculoskeletal applications. Radiographics.

[28] Gennisson JL, Deffieux T, Fink M, Tanter M (2013). Ultrasound elastography: principles and techniques. Diagn Interv Imaging.

[29] Jonkman AH, de Korte CL (2021). Shear wave elastography of the diaphragm: good vibrations?. Am J Respir Crit Care Med.

[30] Moher, D., A. Liberati, J. Tetzlaff, D.G. Altman, and P. Group (2009). Preferred reporting items for systematic reviews and meta-analyses: the PRISMA statement. BMJ.

[31] Mokkink LB, Boers M, van der Vleuten CPM, Bouter LM, Alonso J, Patrick DL (2020). COSMIN Risk of Bias tool to assess the quality of studies on reliability or measurement error of outcome measurement instruments: a Delphi study. BMC Med Res Methodol.

[32] Prinsen CAC, Mokkink LB, Bouter LM, Alonso J, Patrick DL, de Vet HCW (2018). COSMIN guideline for systematic reviews of patient-reported outcome measures. Qual Life Res.

[33] Altman, D.G., Practical statistics for medical research. 1990: CRC press.

[34] Amerijckx C, Goossens N, Pijnenburg M, Musarra F, van Leeuwen DM, Schmitz M (2020). Influence of phase of respiratory cycle on ultrasound imaging of deep abdominal muscle thickness. Musculoskelet Sci Pract.

[35] Baldwin CE, Paratz JD, Bersten AD (2011). Diaphragm and peripheral muscle thickness on ultrasound: intra-rater reliability and variability of a methodology using non-standard recumbent positions. Respirology  (Carlton, Vic.).

[36] Blaney F, English CS, Sawyer T (1998). Sonographic measurement of diaphragmatic displacement during tidal breathing manoeuvres - a reliability study. Aust J Physiother.

[37] Boussuges A, Gole Y, Blanc P, Boussuges A, Gole Y, Blanc P (2009). Diaphragmatic motion studied by m-mode ultrasonography: methods, reproducibility, and normal values. Chest.

[38] Brown C, Tseng SC, Mitchell K, Roddey T (2018). Body position affects ultrasonographic measurement of diaphragm contractility. Cardiopulm Phys Ther J.

[39] Cappellini I, Picciafuochi F, Bartolucci M, Matteini S, Virgili G, Adembri C (2021). Evaluation of diaphragm thickening by diaphragm ultrasonography: a reproducibility and a repeatability study. J Ultrasound.

[40] Harper CJ, Shahgholi L, Cieslak K, Hellyer NJ, Strommen JA, Boon AJ (2013). Variability in diaphragm motion during normal breathing, assessed with B-mode ultrasound. J Orthop Sports Phys Ther.

[41] Marugán-Rubio D, Chicharro JL, Becerro-de-Bengoa-Vallejo R, Losa-Iglesias ME, Rodríguez-Sanz D, Vicente-Campos D (2021). Concurrent Validity and Reliability of Manual Versus Specific Device Transcostal Measurements for Breathing Diaphragm Thickness by Ultrasonography in Lumbopelvic Pain Athletes. Sensors (Basel).

[42] Mohan V, Hashim UF, Md Dom S, Sitilerpisan P, Paungmali A (2017). Paungmali, Reliability of diaphragmatic mobility assessment using a real time ultrasound among non-specific low back pain. Bangladesh J Med Sci.

[43] Nassiri, K., M. Abedi, F.D. Manshadi, A.A. Baghban, and M.H. Meymeh, Comparison of the reliability of sonographic measurements of diaphragm thickness and mobility in individuals with and without pelvic girdle pain. Iranian Red Crescent Med J, 2019. 21(12).

[44] Noh DK, Koh JH, You JS (2016). Inter- and intratester reliability values of ultrasound imaging measurements of diaphragm movement in the thoracic and thoracolumbar curves in adolescent idiopathic scoliosis. Physiother Theory Pract.

[45] Noh DK, Lee JJ, You JH (2014). Diaphragm breathing movement measurement using ultrasound and radiographic imaging: a concurrent validity. Bio-Med Mater Eng.

[46] Oppersma E, Hatam N, Doorduin J, van der Hoeven JG, Marx G, Goetzenich A (2017). Functional assessment of the diaphragm by speckle tracking ultrasound during inspiratory loading. J Appl Physiol  (1985).

[47] Orde SR, Boon AJ, Firth DG, Villarraga HR, Sekiguchi H (2016). Diaphragm assessment by two dimensional speckle tracking imaging in normal subjects. BMC Anesthesiol.

[48] Scarlata S, Mancini D, Laudisio A, Raffaele AI (2019). Reproducibility of diaphragmatic thickness measured by M-mode ultrasonography in healthy volunteers. Respir Physiol Neurobiol.

[49] Scarlata S, Mancini D, Laudisio A, Benigni A, Antonelli Incalzi R (2018). Reproducibility and Clinical Correlates of Supine Diaphragmatic Motion Measured by M-Mode Ultrasonography in Healthy Volunteers. Respiration.

[50] Soilemezi E, Savvidou S, Sotiriou P, Smyrniotis D, Tsagourias M, Matamis D (2020). Tissue doppler imaging of the diaphragm in healthy subjects and critically ill patients. Am J Respir Crit Care Med.

[51] Wallbridge P, Parry SM, Das S, Law C, Hammerschlag G, Irving L (2018). Parasternal intercostal muscle ultrasound in chronic obstructive pulmonary disease correlates with spirometric severity. Sci Rep.

[52] Ziaeifar, M., J. Sarrafzadeh, S. Noorizadeh Dehkordi, A.M. Arab, H. Haghighatkhah, and A. Zendehdel Jadehkenari, Diaphragm thickness, thickness change, and excursion in subjects with and without nonspecific low back pain using B-mode and M-mode ultrasonography. Physiother Theory Pract, 2021: p. 1–11. 10.1080/09593985.2021.1926022 34061721

[53] Marugán-Rubio D, Chicharro JL, Becerro-de-Bengoa-Vallejo R, Losa-Iglesias ME, Rodríguez-Sanz D, Vicente-Campos D (2021). Concurrent Validity and Reliability of Manual Versus Specific Device Transcostal Measurements for Breathing Diaphragm Thickness by Ultrasonography in Lumbopelvic Pain Athletes. Sensors (Basel, Switzerland).

[54] Laghi, F.A., Jr., M. Saad, and H. Shaikh, Ultrasound and non-ultrasound imaging techniques in the assessment of diaphragmatic dysfunction. BMC Pulm Med, 2021. 21(1): p. 85. 10.1186/s12890-021-01441-6 PMC7958108 33722215

[55] Toledo NS, Kodaira SK, Massarollo PC, Pereira OI, Dalmas JC, Cerri GG (2006). Left hemidiaphragmatic mobility: assessment with ultrasonographic measurement of the craniocaudal displacement of the splenic hilum and the inferior pole of the spleen. J Ultrasound Med.

[56] Laveneziana P, Albuquerque A, Aliverti A, Babb T, Barreiro E, Dres M (2019). ERS statement on respiratory muscle testing at rest and during exercise. Eur Respir J.

[57] Mo J, Xu H, Qiang B, Giambini H, Kinnick R, An KN (2016). Bias of shear wave elasticity measurements in thin layer samples and a simple correction strategy. Springerplus.

[58] Sadeghi S, Cortes DH (2020). Measurement of the shear modulus in thin-layered tissues using numerical simulations and shear wave elastography. J Mech Behav Biomed Mater.

[59] Boon AJ, Harper CJ, Ghahfarokhi LS, Strommen JA, Watson JC, Sorenson EJ (2013). Two-dimensional ultrasound imaging of the diaphragm: quantitative values in normal subjects. Muscle Nerve.

[60] Taljanovic MS, Melville DM, Klauser AS, Latt LD, Arif-Tiwari H, Gao L (2015). Advances in lower extremity ultrasound. Curr Radiol Rep.

[61] Thavendiranathan P, Poulin F, Lim KD, Plana JC, Woo A, Marwick TH (2014). Use of myocardial strain imaging by echocardiography for the early detection of cardiotoxicity in patients during and after cancer chemotherapy: a systematic review. J Am Coll Cardiol.

[62] Fabiani I, Pugliese NR, Santini V, Conte L, Di Bello V. Speckle-Tracking Imaging, Principles and Clinical Applications: A Review for Clinical Cardiologists, in Echocardiography in Heart Failure and Cardiac Electrophysiology. 2016.

[63] Dandel M, Hetzer R (2009). Echocardiographic strain and strain rate imaging–clinical applications. Int J Cardiol.

