# Supplementary material for: Reliability and validity of ultrasonography in evaluating the thickness, excursion, stiffness, and strain rate of respiratory muscles in non-hospitalized individuals: a systematic review
Source: BMC Oral Health. 2023 Dec 2;23:959. doi: 10.1186/s12903-023-03558-y (PMC10693145; doi:10.1186/s12903-023-03558-y)
Supplement: Supplementary file 1 — Additional file 1. [file 12903_2023_3558_MOESM1_ESM.docx]

## SUPPLEMENTARY FILES

| **S1. Searching strategy**   \| Step 1 \| reliabilit* or repeatabilit* or reproducibilit* or validit* or validation or responsiveness or temporal change* or change* over time \| \| --- \| --- \| \| Step 2 \| ultrasonograph* or ultrasound* or ultrasonic or sonography or echograph* or shear wave or shear modulus or elastic* modulus or elastogra* or elastic* imaging* \| \| Step 3 \| respiratory muscle* or ventilatory muscle* or breathing muscle* or diaphragm* or intercostal adj3 muscle* or inspiratory adj3 muscle* \| \| Step 4 \| 1 and 2 and 3 \| |
| --- | --- | --- | --- | --- | --- | --- | --- | --- |

| **S2. Updated criteria for good measurement properties**   \| **Measurement property** \| **Rating*** \| **Criteria** \| \| --- \| --- \| --- \| \| Reliability \| ＋ \| ICC or weighted Kappa ≥ 0.70 \| \|  \| ? \| ICC or weighted Kappa not reported \| \| **–** \| ICC or weighted Kappa < 0.70 \| \| Hypotheses testing for construct validity ‡ \| ＋ \| The result is in accordance with the hypothesis^†^ \| \| ? \| No hypothesis defined (by the review team) \| \| **–** \| The result is not in accordance with the hypothesis^†^ \| \| *The COSMIN criteria[1]* \| \| \| \| *Abbreviations: AUC = area under the curve，ICC = intraclass correlation coefficient.* \| \| \| \| ***** *“+” = sufficient, “–” = insufficient, “?” = indeterminate.* \| \| \| \| ^†^ *The results of all studies should be taken together and it should then be decided if 75% of the results are in accordance with the hypotheses.*  ‡ *Generic hypotheses[1]:*  *1. Correlations with (changes in) instruments measuring similar constructs should be ≥0.50.*  *2. Correlations with (changes in) instruments measuring related, but dissimilar constructs should be lower, i.e., 0.30‐0.50.*  *3. Correlations with (changes in) instruments measuring unrelated constructs should be <0.30.*  *4. Meaningful changes between relevant (sub)groups (e.g., patients with expected high vs low levels of the construct of interest)* \| \| \| |
| --- | --- | --- | --- | --- | --- | --- | --- | --- | --- | --- | --- | --- | --- | --- | --- | --- | --- | --- | --- | --- | --- | --- | --- | --- | --- | --- | --- | --- | --- | --- |

| **S3. Definitions of quality levels from adapted GRADE approach**   \| **Quality level** \| **Definition** \| \| --- \| --- \| \| High \| We are very confident that the true measurement property lies close to that of the estimate* of the measurement property \| \| Moderate \| We are moderately confident in the measurement property estimate: the true measurement property is likely to be close to the estimate of the measurement property, but there is a possibility that it is substantially different \| \| Low \| Our confidence in the measurement property estimate is limited: the true measurement property may be substantially different from the estimate of the measurement property \| \| Very low \| We have very little confidence in the measurement property estimate: the true measurement property is likely to be substantially different from the estimate of the measurement property \| \| *The definitions are from adapted GRADE approach in Prinsen et al., 2018[1]* \| \| \| ** Estimate of the measurement property refers to the pooled or summarized result of the measurement property of ClinROMs or PROMs.* \| \| |
| --- | --- | --- | --- | --- | --- | --- | --- | --- | --- | --- | --- | --- | --- | --- |

| **S4. Ultrasound measurement approach**   \| **Target Muscle(s)** \| **Measurement property** \| **Position** \| **Equipment parameters** \| **Transducer location** \| **Movement** \| \| --- \| --- \| --- \| --- \| --- \| --- \| \| Diaphragm \| Thickness:  right[2-4], bilateral[5-9] \| Supine[2, 5, 6], semi-recumbent position[3, 4, 7] or hook-lying position[8, 9]; supine, sitting, and standing[2] \| B-mode, M-mode[7], high-frequency (7-13MHz) linear array probe \| Zone of apposition (ZOA): along the 8^th^-11^th^ ribs between the mid and anterior axillary lines \| At the end of quiet expiration and the end of maximal inspiration; at different breathing volumes[3] \| \| Excursion:  right,[9-13] bilateral,[8, 14, 15] non-specified[16] \| Supine[14, 17, 18], semi-recumbent[12, 13] hook-lying[8, 9], standing[15], sitting[16] \| M-mode, low-frequency (2.5-3.5MHz) curve probe \| subcostal area: left side was located between the mid-axillary and anterior axillary line; right side was located between the anterior axillary line and midclavicular line \| From the end of expiration to the end of maximal inspiration \| \| Stiffness:  right[19-21] \| Supine[20, 21], or semi-recumbent position[19] \| SWE mode, high-frequency (4-15MHz) linear array probe \| ZOA \| At the end of tidal expiration \| \| Strain[10, 12] \| Semi-recumbent position \| Speckle tracking, 2.5-9MHz linear transducer \| ZOA \| At end of expiration, end inspiration \| \| Motion velocity[11] \| Semi-recumbent position \| Tissue doppler, phased array 2-4MHz probe \| Subcostal position between the midclavicular and anterior axillary lines \| Tidal breathing \| \| Intercostal muscles \| Thickness[22] \| Supine \| B-mode, high-frequency (6-14MHz) linear array probe \| 2^nd^-3^rd^ intercostal muscles \| At the end of tidal inspiration \| \| Stiffness[23] \| Supine \| SWE mode, high-frequency (4-15MHz) linear array probe \| T5-T6 right intercostal space along the mid-axillary line \| At the end of tidal breathing \| \| Abdominal muscles (TrA and IO) \| Thickness[24] \| Standing \| B-mode, high-frequency (6-13MHz) linear array probe \| 2.5cm medially of the mid-axillary line and halfway between the lowest rib and ilium \| At end of quiet expiration, maximal inspiration, and maximal expiration \|   *Abbreviations: TrA= transverse abdominals; IO= internus obliquus; ZOA=zone of apposition; SWE=shear wave elastography* |
| --- | --- | --- | --- | --- | --- | --- | --- | --- | --- | --- | --- | --- | --- | --- | --- | --- | --- | --- | --- | --- | --- | --- | --- | --- | --- | --- | --- | --- | --- | --- | --- | --- | --- | --- | --- | --- | --- | --- | --- | --- | --- | --- | --- | --- | --- | --- | --- | --- | --- |

| **S5. Quality assessments and level of evidence of using ultrasonography measurements for respiratory muscles in separated populations – Reliability**    *Abbreviations: TrA= Transverse abdominals; IO= internus obliquus; LBP = low back pain; AIS= adolescent idiopathic scoliosis; COPD= chronic obstructive pulmonary disease; Not sure=not sure time interval* |
| --- |

**References**

1. Prinsen CAC, Mokkink LB, Bouter LM, Alonso J, Patrick DL, de Vet HCW, Terwee CB. COSMIN guideline for systematic reviews of patient-reported outcome measures. Qual Life Res. 2018;27(5):1147-57.

2. Brown C, Tseng SC, Mitchell K, Roddey T. Body Position Affects Ultrasonographic Measurement of Diaphragm Contractility. Cardiopulm Phys Ther J. 2018;29(4):166-72.

3. Baldwin CE, Paratz JD, Bersten AD. Diaphragm and peripheral muscle thickness on ultrasound: intra-rater reliability and variability of a methodology using non-standard recumbent positions. Respirology (Carlton, Vic). 2011;16(7):1136-43.

4. Scarlata S, Mancini D, Laudisio A, Raffaele AI. Reproducibility of diaphragmatic thickness measured by M-mode ultrasonography in healthy volunteers. Respiratory physiology & neurobiology. 2019;260:58-62.

5. Marugán-Rubio D, Chicharro JL, Becerro-de-Bengoa-Vallejo R, Losa-Iglesias ME, Rodríguez-Sanz D, Vicente-Campos D, et al. Concurrent Validity and Reliability of Manual Versus Specific Device Transcostal Measurements for Breathing Diaphragm Thickness by Ultrasonography in Lumbopelvic Pain Athletes. Sensors (Basel). 2021;21(13).

6. Harper CJ, Shahgholi L, Cieslak K, Hellyer NJ, Strommen JA, Boon AJ. Variability in diaphragm motion during normal breathing, assessed with B-mode ultrasound. J Orthop Sports Phys Ther. 2013;43(12):927-31.

7. Cappellini I, Picciafuochi F, Bartolucci M, Matteini S, Virgili G, Adembri C. Evaluation of diaphragm thickening by diaphragm ultrasonography: a reproducibility and a repeatability study. Journal of ultrasound. 2021;24(4):411-6.

8. Ziaeifar M, Sarrafzadeh J, Noorizadeh Dehkordi S, Arab AM, Haghighatkhah H, Zendehdel Jadehkenari A. Diaphragm thickness, thickness change, and excursion in subjects with and without nonspecific low back pain using B-mode and M-mode ultrasonography. Physiother Theory Pract. 2021:1-11.

9. Nassiri K, Abedi M, Manshadi FD, Baghban AA, Meymeh MH. Comparison of the reliability of sonographic measurements of diaphragm thickness and mobility in individuals with and without pelvic girdle pain. Iranian Red Crescent Medical Journal. 2019;21(12).

10. Oppersma E, Hatam N, Doorduin J, van der Hoeven JG, Marx G, Goetzenich A, et al. Functional assessment of the diaphragm by speckle tracking ultrasound during inspiratory loading. J Appl Physiol (1985). 2017;123(5):1063-70.

11. Soilemezi E, Savvidou S, Sotiriou P, Smyrniotis D, Tsagourias M, Matamis D. Tissue Doppler Imaging of the Diaphragm in Healthy Subjects and Critically Ill Patients. Am J Respir Crit Care Med. 2020;202(7):1005-12.

12. Orde SR, Boon AJ, Firth DG, Villarraga HR, Sekiguchi H. Diaphragm assessment by two dimensional speckle tracking imaging in normal subjects. BMC anesthesiology. 2016;16(1):43.

13. Mohan V, Hashim UF, Md Dom S, Sitilerpisan P, Paungmali A. Reliability of diaphragmatic mobility assessment using a real time ultrasound among non-specific low back pain. Bangladesh Journal of Medical Science. 2017;16(3):443-7.

14. Noh DK, Lee JJ, You JH. Diaphragm breathing movement measurement using ultrasound and radiographic imaging: a concurrent validity. Bio-medical materials and engineering. 2014;24(1):947-52.

15. Boussuges A, Gole Y, Blanc P, Boussuges A, Gole Y, Blanc P. Diaphragmatic motion studied by m-mode ultrasonography: methods, reproducibility, and normal values. CHEST. 2009;135(2):391-400.

16. Blaney F, English CS, Sawyer T. Sonographic measurement of diaphragmatic displacement during tidal breathing manoeuvres - a reliability study. Aust J Physiother. 1998;44(1):41-3.

17. Scarlata S, Mancini D, Laudisio A, Benigni A, Antonelli Incalzi R. Reproducibility and Clinical Correlates of Supine Diaphragmatic Motion Measured by M-Mode Ultrasonography in Healthy Volunteers. Respiration; international review of thoracic diseases. 2018;96(3):259-66.

18. Noh DK, Koh JH, You JS. Inter- and intratester reliability values of ultrasound imaging measurements of diaphragm movement in the thoracic and thoracolumbar curves in adolescent idiopathic scoliosis. Physiother Theory Pract. 2016;32(2):139-43.

19. Bachasson D, Dres M, Niérat MC, Gennisson JL, Hogrel JY, Doorduin J, Similowski T. Diaphragm shear modulus reflects transdiaphragmatic pressure during isovolumetric inspiratory efforts and ventilation against inspiratory loading. J Appl Physiol (1985). 2019;126(3):699-707.

20. Flattres A, Aarab Y, Nougaret S, Garnier F, Larcher R, Amalric M, et al. Real-time shear wave ultrasound elastography: a new tool for the evaluation of diaphragm and limb muscle stiffness in critically ill patients. Critical Care. 2020;24(1).

21. Xu JH, Wu ZZ, Tao FY, Zhu ST, Chen SP, Cai C, et al. Ultrasound Shear Wave Elastography for Evaluation of Diaphragm Stiffness in Patients with Stable COPD: A Pilot Trial. J Ultrasound Med. 2021;40(12):2655-63.

22. Wallbridge P, Parry SM, Das S, Law C, Hammerschlag G, Irving L, et al. Parasternal intercostal muscle ultrasound in chronic obstructive pulmonary disease correlates with spirometric severity. Scientific reports. 2018;8(1):15274.

23. Pietton R, David M, Hisaund A, Langlais T, Skalli W, Vialle R, Vergari C. Biomechanical Evaluation of Intercostal Muscles in Healthy Children and Adolescent Idiopathic Scoliosis: A Preliminary Study. Ultrasound in medicine & biology. 2021;47(1):51-7.

24. Amerijckx C, Goossens N, Pijnenburg M, Musarra F, van Leeuwen DM, Schmitz M, Janssens L. Influence of phase of respiratory cycle on ultrasound imaging of deep abdominal muscle thickness. Musculoskelet Sci Pract. 2020;46:102105.
